# Small molecule disruption of G protein βγ subunit signaling reprograms human macrophage phenotype and prevents autoimmune myocarditis in rats

**DOI:** 10.1371/journal.pone.0200697

**Published:** 2018-07-19

**Authors:** Vengadeshprabhu Karuppagounder, Anamika Bajpai, Shu Meng, Somasundaram Arumugam, Remya Sreedhar, Vijayasree V. Giridharan, Ashrith Guha, Arvind Bhimaraj, Keith A. Youker, Suresh S. Palaniyandi, Harry Karmouty-Quintana, Fadia Kamal, Kara L. Spiller, Kenichi Watanabe, Rajarajan A. Thandavarayan

**Affiliations:** 1 Department of Clinical Pharmacology, Faculty of Pharmaceutical Sciences, Niigata University of Pharmacy and Applied Life Sciences, Niigata, Japan; 2 Department of Orthopaedic and Rehabilitation, Penn State college of medicine, Hershey, Pennsylvania, United States of America; 3 Biomaterials and Regenerative Medicine Laboratory, School of Biomedical Engineering, Science, and Health Systems, Drexel University, Philadelphia, Pennsylvania, United States of America; 4 Department of Biochemistry and Molecular Biology, Houston Medical School, University of Texas, Houston, United States of America; 5 Department of Psychiatry and Behavioral Sciences, Translational Psychiatry Program, McGovern Medical School, Houston, Texas, United States of America; 6 Houston Methodist DeBakey Heart and Vascular Center, Houston Methodist Hospital, Houston, Texas, United States of America; 7 Division of Hypertension and Vascular Research, Henry Ford Health System, Detroit, Michigan, United States of America; University of Alabama at Birmingham, UNITED STATES

## Abstract

The purpose of this study was to determine whether blocking of G protein βγ (Gβγ) signaling halts heart failure (HF) progression by macrophage phenotype manipulation. Cardiac Gβγ signaling plays a crucial role in HF pathogenesis. Previous data suggested that inhibiting Gβγ signaling reprograms T helper cell 1 (Th1) and Th2 cytokines, suggesting that Gβγ might be a useful drug target for treating HF. We investigated the efficacy of a small molecule Gβγ inhibitor, gallein, in a clinically relevant, experimental autoimmune myocarditis (EAM) model of HF as well as in human macrophage phenotypes in vitro. In the myocardium of HF patients, we observed that G protein coupled receptor kinase (GRK)2 levels were down-regulated compared with healthy controls. In rat EAM, treatment with gallein effectively improved survival and cardiac function, suppressed cardiac remodeling, and further attenuated myocardial protein expression of GRK2 as well as high mobility group box (HMGB)1 and its cascade signaling proteins. Furthermore, gallein effectively inhibited M1 polarization and promoted M2 polarization *in vivo* in the EAM heart and *in vitro* in human monocyte-derived macrophages. Taken together, these data suggest that the small molecule Gβγ inhibitor, gallein, could be an important pharmacologic therapy for HF as it can switch the phenotypic reprogramming from M1 to M2 phenotype in a rat model of EAM heart and in human macrophages.

## Introduction

Heart failure (HF) is a leading cause of morbidity and mortality worldwide. Increasing evidence suggests that G protein βγ (Gβγ) signaling plays an important role in HF pathophysiology [1–5]. In myocardial cells, ligand binding to G protein coupled receptors (GCPRs) catalyzes the exchange of tightly bound GDP for GTP on the Gα subunit, liberating it from the Gβγ subunits [2]. Dissociation of the Gα and Gβγ subunits allows each to directly interact with downstream effector proteins. The Gβγ subunits interact with G protein coupled receptor kinase (GRK)2, triggering chronic desensitization of β-adrenergic receptor (β-AR) and leading to HF [6, 7]. In addition, GRK2 levels were significantly elevated in an animal model of HF and in human patients [1, 8]. Other studies reported that enhancing Gβγ-GRK2 interactions by cardiac-targeted overexpression of GRK2 can directly cause HF in preclinical studies; in contrast genetic ablation of GRK2 has generally proven to be cardioprotective [9–11].

Inflammation and autoimmunity contribute to many cardiovascular diseases (CVD) [12]. Gβγ signaling activates signal transducer and activator of transcription (STAT)4 and interferon (IFN)γ in CD4+T cells, which triggers T helper cells (Th1) and pro-inflammatory (M1) macrophage phenotype differentiation in autoimmune diseases [13]. The classical pro-inflammatory or so-called M1 macrophages are activated by inflammatory stimuli such as IFNγ, and secrete large amounts of proinflammatory mediators, which are associated with cardiac damage [14]. In contrast, the M2 designation has been applied to alternatively activated macrophages; this phenotype is divided into at least two subtypes: M2a and M2c, which are stimulated by interleukin (IL)-4/IL-13 and IL-10, respectively. The M2 phenotype has been shown to improve cardiac repair after inflammation or injury, although the mechanisms are poorly understood [15].

These previous studies suggest that small molecule inhibition of Gβγ signaling may be useful for the treatment of HF. Importantly, β blockers are standard therapy for human HF patients. Previously our group reported that the β blocker carvedilol effectively blocked β-adrenergic receptors (ARs) in HF models [16], however, it did not specifically inhibit Gβγ subunits. Interestingly, gallein is a cell-permeable xanthene compound that binds to Gβγ with high affinity and inhibits Gβγ; halts HF progression in a murine transverse aortic constriction model [1]. These results suggest that targeting Gβγ might provide an effective way to block signaling from the multiple GPCRs that can trigger M1 macrophage polarization, which may have an effect on HF. On the basis of these studies, we hypothesized that gallein would ameliorate cardiac dysfunction and inflammation by reprogramming M1 to M2 macrophage polarization in the heart of experimental autoimmune myocarditis (EAM) rats and *in vitro* with human macrophages.

## Materials and methods

### Materials

Gallein pure drug was purchased from Sigma Aldrich, Japan. All chemicals and reagents were purchased from Sigma (Tokyo, Japan), unless otherwise mentioned.

### Human samples

Fresh human heart samples were obtained directly from the surgeon at the time of human heart transplantation at the Houston Methodist DeBakey Heart and Vascular center, Houston Methodist Hospital, Houston, Texas. Normal hearts were obtained from donor hearts that were not used for transplantation and were collected and stored in the same manner. All tissues were collected under an approved protocol (IRB(3N)0511-0100) by the Houston Methodist Hospital Institutional Review Board. This is a HIPAA-compliant, IRB approved study with a waiver of consent. All tissues used were from de-identified samples. Scar-free, reddish left ventricle (LV) apex samples (≈2 g) were immediately dissected and tissue was freeze clamped in liquid nitrogen for RNA and protein analysis within 1 to 2 minutes following the hand off. Additional samples were paraffin-embedded for histology.

### Animal studies experimental design

Seven week-old male Lewis rats were purchased from Charles River Japan Inc (Kanagawa, Japan) and were maintained individually (because of their aggressive behavior) under standard conditions (temperature 23 ± 1°C, humidity 50–60%, 12:12-h light-dark cycle, lights on at 7:00 a.m.), with food in the form of dry pellets and tap water available ad libitum throughout the study. After 1 week, the rats (8 weeks of age) were randomly divided into four groups and the first group served as control (Normal, n = 4). To induce EAM, the other rats were injected in the footpads with antigen-adjuvant emulsion in accordance with a procedure described previously [17]. In brief, porcine cardiac myosin was dissolved in phosphate buffered saline at 5 mg/mL and emulsified with an equal volume of complete Freund’s adjuvant with 11 mg/mL Mycobacterium tuberculosis H37RA (Difco Lab., Detroit, MI). EAM in rats was induced by immunization with 0.1 mL of emulsion once by subcutaneous injection into the rear footpads (0.1 mL to each footpad). The morbidity of EAM is 100% in rats immunized by this procedure [12]. After myosin injection, rats were randomly divided into three groups and each received either vehicle (drinking water) alone (EAM, n = 5) or Gallein 10 mg/kg/day (EAM+G10, n = 5), for 21 days. By the end of the study period, the rats’ body weights were measured, and rats were anesthetized with 2% halothane and subjected to echocardiography. After this measurement, blood was drawn by cardiac puncture and heart tissue was harvested for Western blotting and histological analysis. The experiment was performed in accordance with the national guidelines and approved by the animal care committee of Niigata University of Pharmacy and Applied Life Sciences (Approval no: H27040), Niigata, Japan.

### Transthoracic echocardiographic analysis

By the end of the study, the left ventricular dimension in diastole (LVDd) and in systole (LVDs), and percentage of ejection fraction (EF) and fractional shortening (FS) were measured using M-mode measurements [18]. After the echocardiographic analysis, rats were sacrificed and their heart weights (HW) were measured.

### Histopathological analysis

Half of each heart was immediately snap frozen in liquid nitrogen for subsequent protein extraction assays. The remaining excised heart tissue was cut into 2 mm transverse slices and fixed in 10% formalin. Sections of 3–5 μm thickness were stained with hematoxylin & eosin (H&E), toluidine blue (TB) and Masson’s trichrome (MT) for histological examination [19, 20].

### Western blotting

Protein lysates were prepared from cardiac tissue as described previously. The total protein concentration in the samples were measured by the bicinchoninic acid method [20]. For Western blotting experiments, 50 μg of total protein was loaded and proteins were separated by SDS-PAGE (200 V for 40 min) and electrophorectically transferred to nitrocellulose filters. Filters were blocked with 5% bovine serum albumin in Tris buffered saline (TBS), (20 mM Tris, pH 7.6, 137 mM NaCl) with 0.1% Tween 20, washed and then incubated individually with the following antibodies: Antibodies against GRK2, high mobility group box protein (HMGB)1, toll-like receptor (TLR)4, extracellular regulated kinase (ERK)1/2, tumor necrosis factor (TNF)α, IFNγ, and cyclooxygenase (COX)2. Glycealdehyde-3-phosphate dehydrogenase (GAPDH) served as a control protein [21]. All the antibodies were purchased from Santa Cruz Biotechnology, Inc., (Santa Cruz, CA, USA) or Cell Signaling Technology, Inc. (Danvers, MA, USA) and used at a dilution of 1:1000. After washing for three times with TBST, the membranes were incubated with appropriate horseradish peroxidase (HRP)-conjugated secondary antibodies for 1 h at room temperature. Then, the membranes were washed three times with TBST and then developed using a chemiluminescence detection system (ImmunoStar LD, Wako Pure Chemical, Osaka, Japan). The blots were scanned with the Image Studio Digits ver. 4 (Superior Street, Lincoln, Nebraska, USA).

### Immunohistochemical (IHC) analysis

Formalin-fixed, paraffin-embedded cardiac tissue sections were used for IHC staining. After deparaffinization and hydration, the slides were washed in TBS (TBS; 10 mM/1 Tris HCl, 0.85% NaCl, pH 7.2). Endogenous peroxidase activity was quenched by incubating the slides in 0.3% H_2_O_2_ in methanol. The slides were blocked with either 10% goat serum or rabbit serum for 1 h at room temperature and washed three times for 5 min each. After overnight incubation with primary antibody (human: GRK2; rats: cluster of differentiation (CD)36, CD80, CD68, CD163 and IL10; Santa Cruz Biotechnology, Inc., CA, USA, diluted 1:100), at 4°C, the slides were further rinsed in TBS and then HRP-conjugated secondary antibody was added and the slides were further incubated at room temperature for 45 min. The slides were rinsed in TBS and incubated with diaminobenzidine tetra hydrochloride as the substrate and counterstained with hematoxylin. Brown colored immunopositive CD80, CD68, CD163, CD36, and IL10 cells were counted using 40X objective [14, 22].

### Preparation and characterization of polarized human macrophages in vitro

Freshly isolated primary monocytes were purchased from the University of Pennsylvania Human Immunology Core. Human monocytes were differentiated and polarized into macrophages phenotypes (M0, M1, M2a, M2c) as per previously established 7-day protocols [23]. Briefly, primary human monocytes were cultured at 37°C and 5% CO2 in 24 well ultra-low attachment plates (Corning) for five days at a density of 1.0×10^6^ cells/ml in complete media (RPMI media supplemented with 10% heat-inactivated human serum, 1% penicillin-streptomycin, and 20 ng/ml macrophage colony stimulating factor (MCSF)) for 5 days, with a media change on day 3, to differentiate monocytes into M0 macrophages. M0 macrophages were polarized for the next 2 days by culturing at 1.0×10^6^ cells/ml in complete media with 100 ng/ml IFNγ (Peprotech, Rocky Hill, NJ) and 100 ng/ml lipopolysaccharide (LPS, Sigma Aldrich) for M1, 40 ng/ml IL-4 and 20 ng/ml IL-13 (Peprotech, Rocky Hill, NJ) for M2a and 40 ng/mL IL-10 (Peprotech, Rocky Hill, NJ) for M2c phenotype. Unactivated macrophages (M0) were also cultured over the same time period in the absence of polarizing cytokines.

### Effects of gallein on human macrophage phenotype

On day 5 of macrophage culture (M0), gallein was added at 10 μM per 1.0×10^6^ cells at the same time as polarization into the phenotypes described above (M0, M1, M2a, M2c). Macrophages that were not treated with gallein were considered as controls (n = 3 for each group). After 2 days of polarization, macrophages were collected by scraping and stored in lysis buffer (RNAqueous-Micro, Life Technologies) at -80°C.

### RNA extraction, complementary cDNA synthesis, and quantitative real time reverse transcription polymerase chain reaction (qRT-PCR)

Total RNA was extracted from cells using RNAqueous-Micro kit (Life Technologies) according to the manufacturer's instructions. DNase treatment was performed using DNase I amplification grade (Invitrogen, Carlsbad, CA, USA). Afterwards, cDNA was synthesized using high-capacity cDNA reverse transcription kit (Applied Biosystems, Foster City, CA, USA) and samples were stored at -80°C. Expression of multiple markers of macrophage phenotype were tested using qRT-PCR with GAPDH as a house keeping gene, using 20ng RNA per reaction, as previously described [23, 24]. The following macrophage phenotypes related genes were used for qRT-PCR: M1 phenotype- vascular endothelial growth factor (VEGF), C-C chemokine receptor (CCR)7, CD80, IL1B; M2a phenotype- platelet derived growth factor (PDGFB), tissue inhibitor of metalloproteinase (TIMP)3, mannose receptor C type 1 (MRC1); M2c phenotype- Versican (VCAN), macrophage with collagenous structure (MARCO), CD163 (Table 1. The expression of target genes was normalized to the housekeeping gene GAPDH, and then to the unactivated M0 phenotype).

**Table 1:** List of sequence specific primer used for q-PCR.

Genes name	Primers sequence (5' to 3')
GAPDH-F	AAGGTGAAGGTCGGAGTCAAC
GAPDH-R	GGGGTCATTGATGGCAACAATA
CD80-F	AAACTCGCATCTACTGGCAAA
CD80-R	GGTTCTTGTACTCGGGCCATA
CCR7-F	TGAGGTCACGGACGATTACAT
CCR7-R	GTAGGCCCACGAAACAAATGAT
IL1b-F	ATGATGGCTTATTACAGTGGCAA
IL1b-R	GTCGGAGATTCGTAGCTGGA
VEGF-F	AGGGCAGAATCATCACGAAGT
VEGF-R	AGGGTCTCGATTGGATGGCA
CD206-F	AAGGCGGTGACCTCACAAG
CD206-R	AAAGTCCAATTCCTCGATGGTG
PDGF-F	CTCGATCCGCTCCTTTGATGA
PDGF-R	CGTTGGTGCGGTCTATGAG
TIMP3-F	ACCGAGGCTTCACCAAGATG
TIMP3-R	CATCATAGACGCGACCTGTCA
CD163-F	TTTGTCAACTTGAGTCCCTTCAC
CD163-R	TCCCGCTACACTTGTTTTCAC
MARCO-F	CAGCGGGTAGACAACTTCAC
MARCO-R	TTGCTCCATCTCGTCCCATAG
VCAN-F	GCAAGTGATGCGGGTCTTTAC
VCAN-R	TTGCCGCCCTGTAGTGAAAC

### Statistical analysis

Data are presented as mean ± SEM and were analyzed using one way analysis of variance (ANOVA) followed by Tukey’s multiple comparison test or student’s t-test when appropriate. A value of p<0.05 was considered statistically significant. For statistical analysis, GraphPad Prism 5 and 6 software (San Diego, CA) were used.

## Results

### Human failing heart shows increased grk2 expression

**Fig 1:**
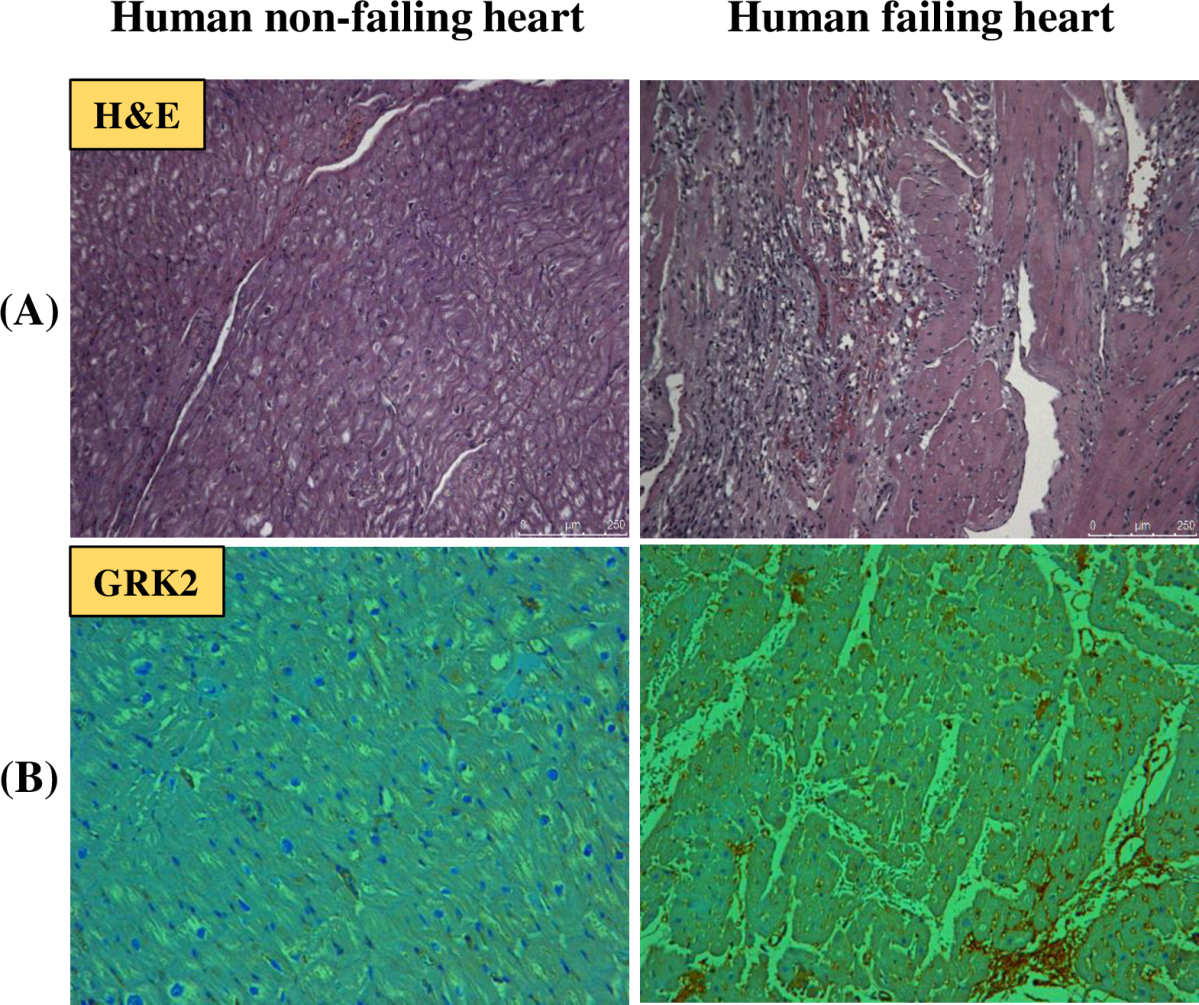
Cardiac expression of GRK2 in patients with HF. (A) Haematoxylin and eosin (H&E) staining and (B) GRK2 IHC staining of human cardiac tissue (10X).

Previous reports have shown that an increase in the inflammatory response in the myocardium after pressure overload corroborated with increased Gβγ-GRK2 interactions and higher GRK-2 expression levels, leading to HF [1]. In the present study, to determine the effect of HF on GRK-2 expression, cardiac biopsies were collected from the LV of patients who suffered from HF with ischemia at the Houston Methodist DeBakey Heart & Vascular Center, Houston, Texas. Histological evaluation of human failing heart tissues showed increased infiltration of inflammatory cells into the myocardium (Fig 1A), concomitant with increased GRK-2 expression as compared to non-failing heart tissue sections (Fig 1B).

### Effect of gallein on morphometric and echocardiographic parameters in EAM rats

**Fig 2:**
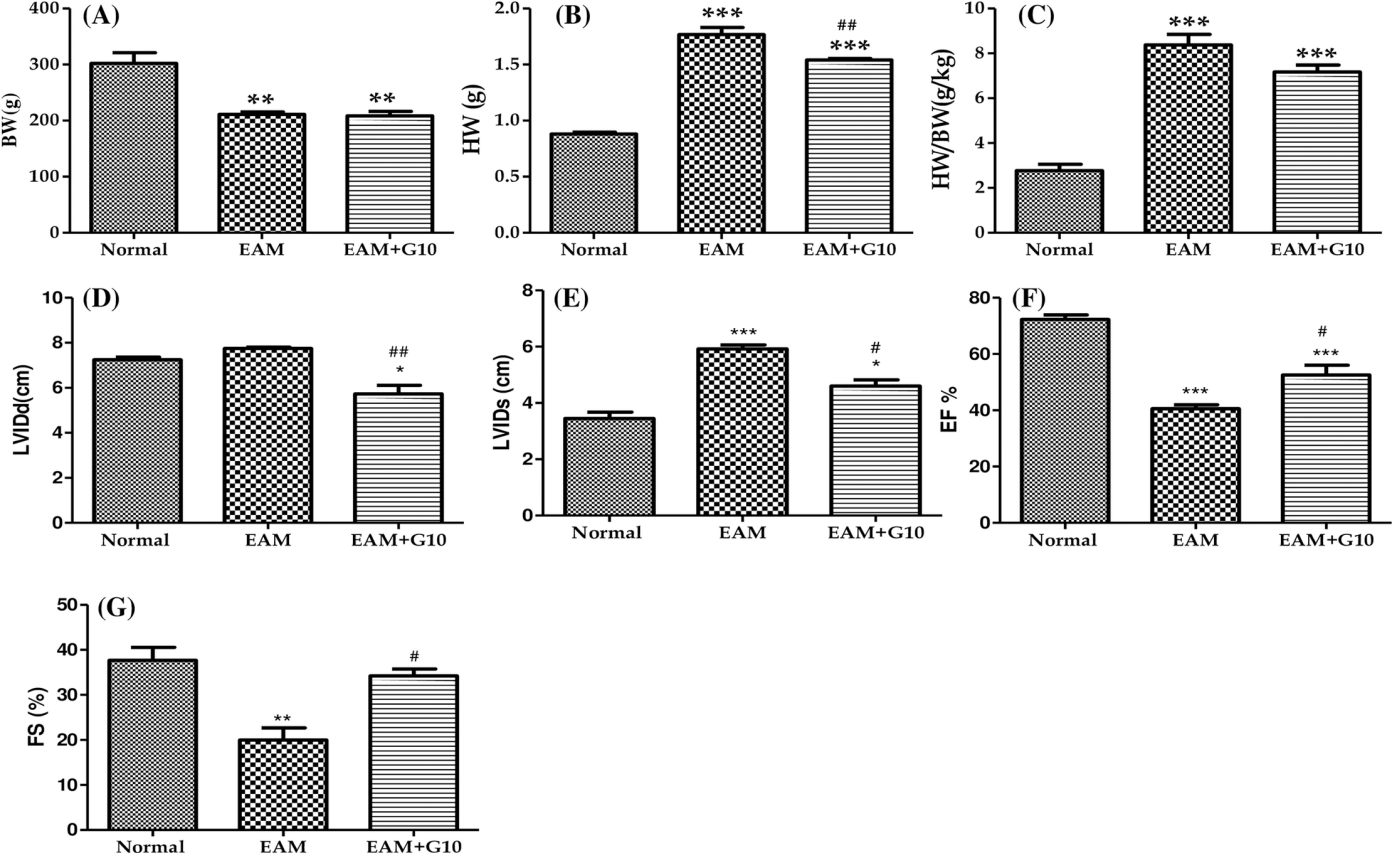
Effect of gallein on BW, HW and echocardiographic parameters. **(A-G)** BW, body weight; HW, heart weight; LVDd, left ventricular dimension in diastole; LVDs, left ventricular dimension in systole; FS, fractional shortening; EF, ejection fraction; group Normal, age matched untreated rats; group EAM, rats with experimental autoimmune myocarditis treated with vehicle; group EAM+G10, rats with EAM treated with gallein given orally at 10 mg/kg/day. ***p<0.001, **p<0.01 and *p<0.05 vs Normal; ^##^p<0.01 and ^#^p<0.05 vs EAM. Results are presented as mean ± SEM, n = 4–5.

EAM rats had significantly lower mean body weights (BW) than age-matched normal rats (Fig 2A, p<0.001). Gallein treatment did not alter or prevent the reduction of BW of EAM rats. HW and the ratio of HW/BW were significantly increased in EAM group rats than in normal rats (Fig 2B and 2C). Gallein treatment attenuated these increases in HW and the ratio of HW/BW when compared with vehicle-treated EAM rats.

Echocardiographic studies of EAM group rats showed significantly increased LVDd and LVDs as well as reduced EF (40.54±1.445% vs 72.32±1.57%, p<0.001) and FS (20±2.67 vs 37.67±2.90%, p<0.01). These data indicate impairment of cardiac function in the EAM group compared with control group. Treatment with gallein significantly decreased LVDs increased EF and FS when compared with those of vehicle-treated EAM rats (Fig 2E–2G).

### Effect of gallein on myocardial histological changes

**Fig 3:**
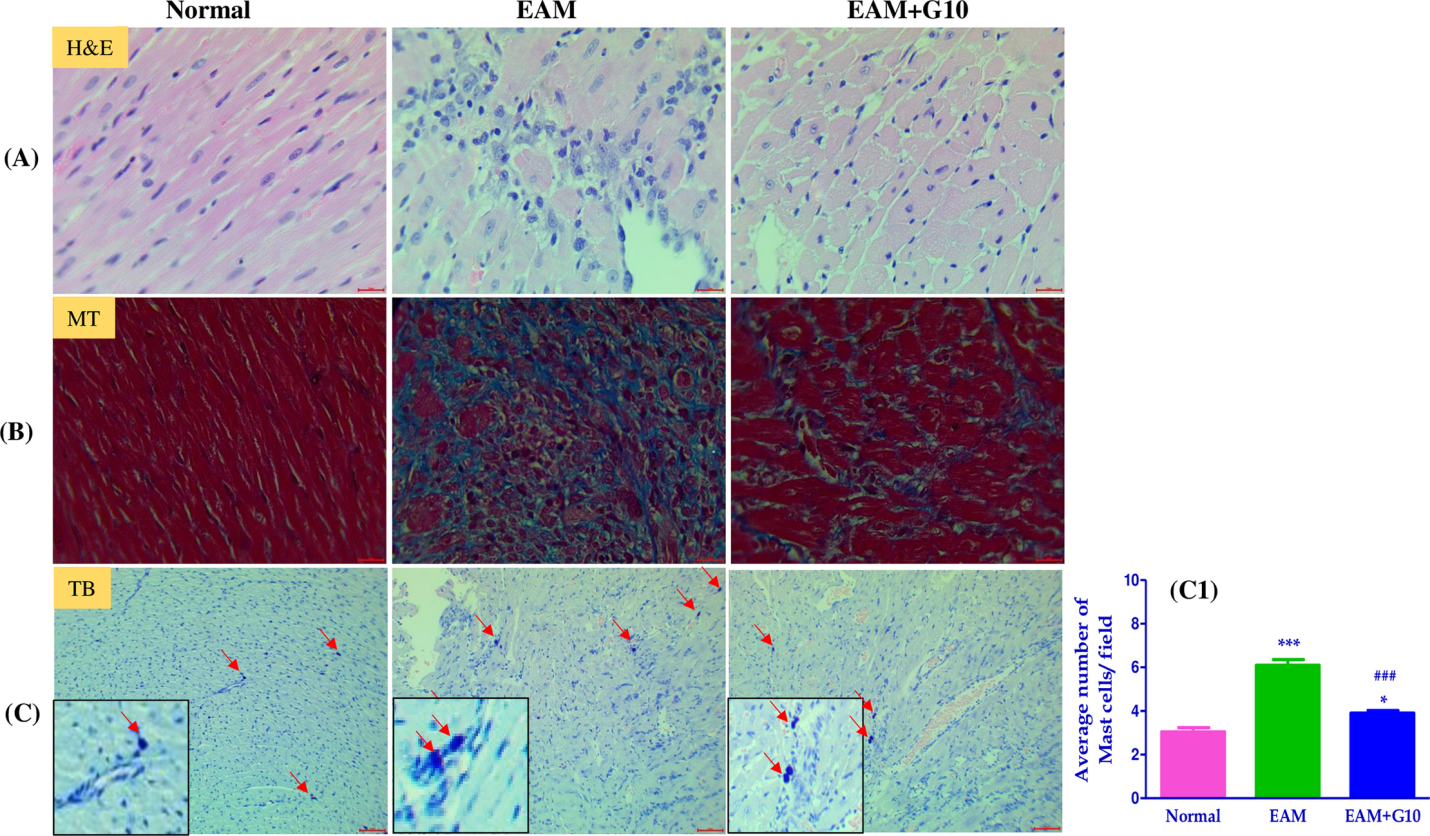
Effect of gallein on myocardial histological changes. (A) H&E staining of left ventricular sections depicting infiltration of inflammatory cells, interstitial edema, vacuolization, and degeneration of cardiac fibers. (B) Masson’s trichrome (MT) staining for fibrosis (blue area) in the cross sectional tissue sections of left ventricle. (C-C1) Toluidine blue (TB) staining for mast cells of the cross sectional slices of heart and their quantification. Scale bar = 20 μm. Each bar represents mean ± SEM, n = 4–5. Normal, age matched normal rats; EAM, rats with experimental autoimmune myocarditis treated with vehicle; EAM+G10, rats with EAM treated with gallein (10 mg/kg/day). ***p<0.001 and *p<0.05 vs Normal; ^###^p<0.001 vs EAM.

H&E and MT staining of cardiac sections revealed severe inflammatory cellular infiltration, damaged cellular organization and interstitial collagen deposition in the heart of EAM rats (Fig 3A–3C). In contrast, gallein-treated EAM rats showed reduction in these myocardial changes compared with the vehicle-treated EAM group. In addition, TB staining showed that the number of inflammatory mast cells were significantly increased in the heart of rats with EAM. Treatment with gallein significantly suppressed mast cells in EAM rats compared with vehicle treated EAM rats (Fig 3C and 3C1).

### Gallein inhibits grk2 expression in myosin-induced EAM

**Fig 4:**
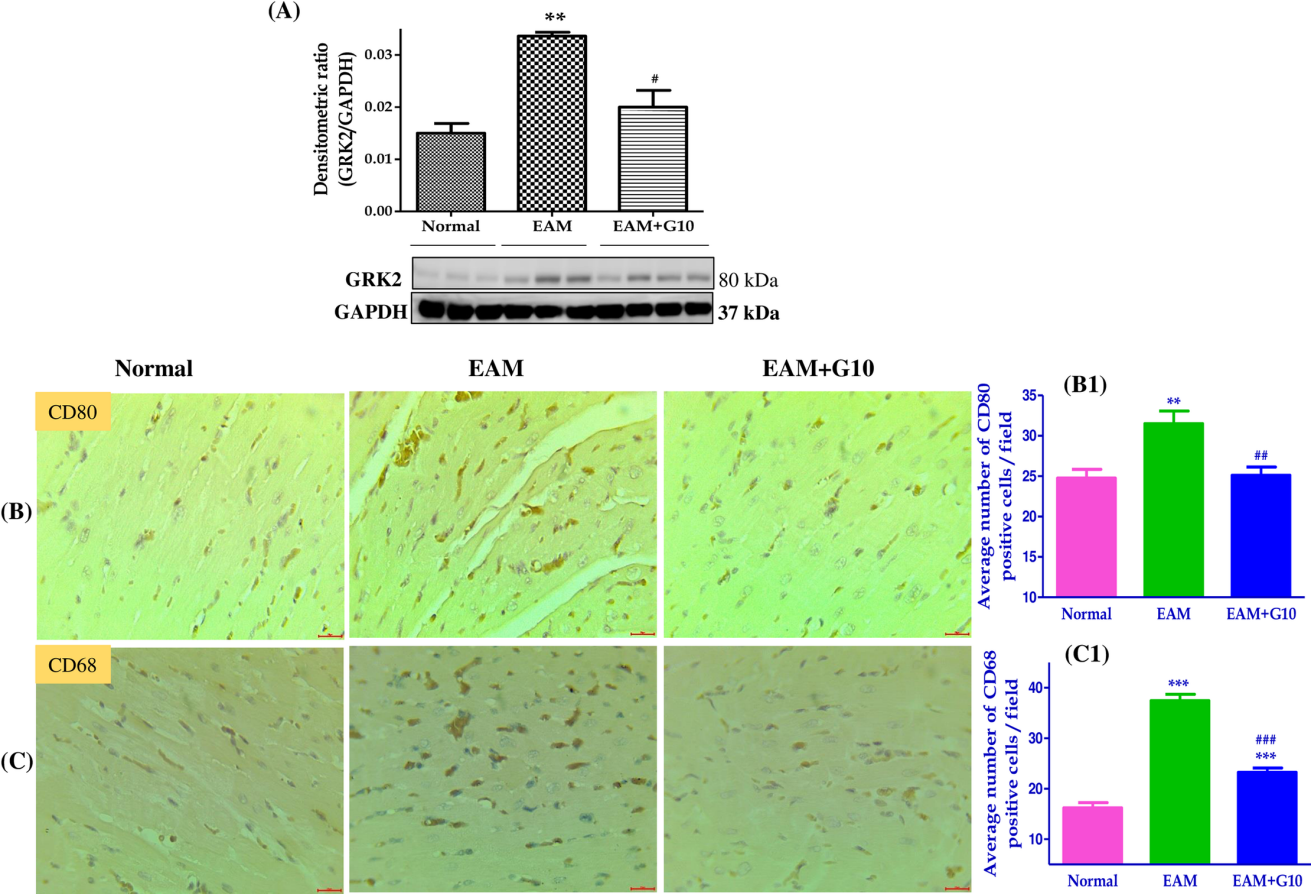
Gallein inhibits GRK2, CD80 and CD68 expression in myosin induced EAM rat heart. Western blots show specific band for the expression of (A) GRK2 (ratio with GAPDH). IHC staining for (B-B1) CD80 and (C-C1) CD68 positive cells and their quantification. Scale bar = 20 μm. Each bar represents mean ± SEM, n = 4–5. Normal, age matched normal rats; EAM, rats with experimental autoimmune myocarditis treated with vehicle; EAM+G10, rats with EAM treated with gallein. ***p<0.001, and **p<0.01 vs Normal; ^###^p<0.001, ^##^p<0.01 vs EAM.

Consequently, we examined whether gallein treatment would inhibit GRK2 expression in myosin induced EAM rat myocardium. We found that the expression of GRK2 was significantly upregulated in the heart of EAM rats compared to normal controls, whereas gallein treatment significantly downregulated this expression (Fig 4A).

### Gallein suppressed m1-like macrophage phenotype in myosin induced EAM rats

**Fig 5:**
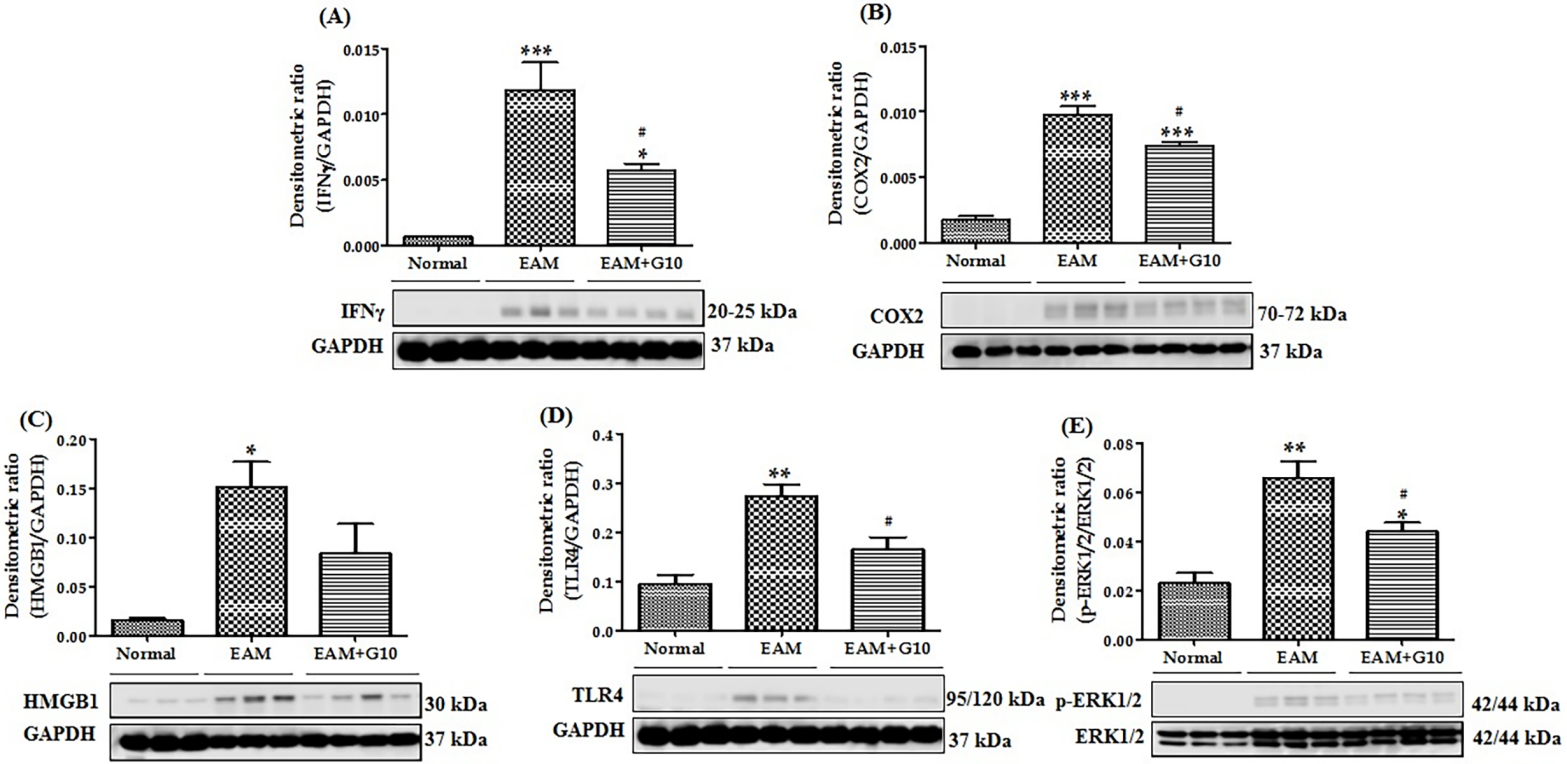
Small molecule Gβγ-GRK2 inhibitor gallein suppressed M1-like macrophage phenotype marker expression in the hearts of EAM rats. Western blots show specific bands for the expression of (A-E) IFNγ, COX2, HMGB1, TLR4 (ratio with GAPDH) and ERK1/2 (ratio with total ERK1/2). Each bar represents mean ± SEM, n = 3–4. Normal, age matched normal rats; EAM, rats with experimental autoimmune myocarditis treated with vehicle; EAM+G10, rats with EAM treated with gallein. ***p<0.001, **p<0.01 and *p<0.05 vs Normal; ^#^p<0.05 vs EAM.

As shown in Fig 4B and 4B1 and 4C and 4C1, IHC examination revealed that M1 phenotype marker CD80 and CD68 positive cells were increased in myosin-treated EAM rats, whereas treatment with gallein significantly decreased their levels in the hearts of EAM rats. Next, we examined M1 phenotype marker expressions in the cardiac tissue by Western blot. The protein expression of IFNγ, COX2, HMGB1, TLR4, ERK1/2 were significantly upregulated in EAM rats compared with those in normal rats and these changes were dramatically suppressed by gallein treatment (Fig 5A–5E).

### Effect of gallein on m2-like macrophage phenotype in myosin induced EAM

**Fig 6:**
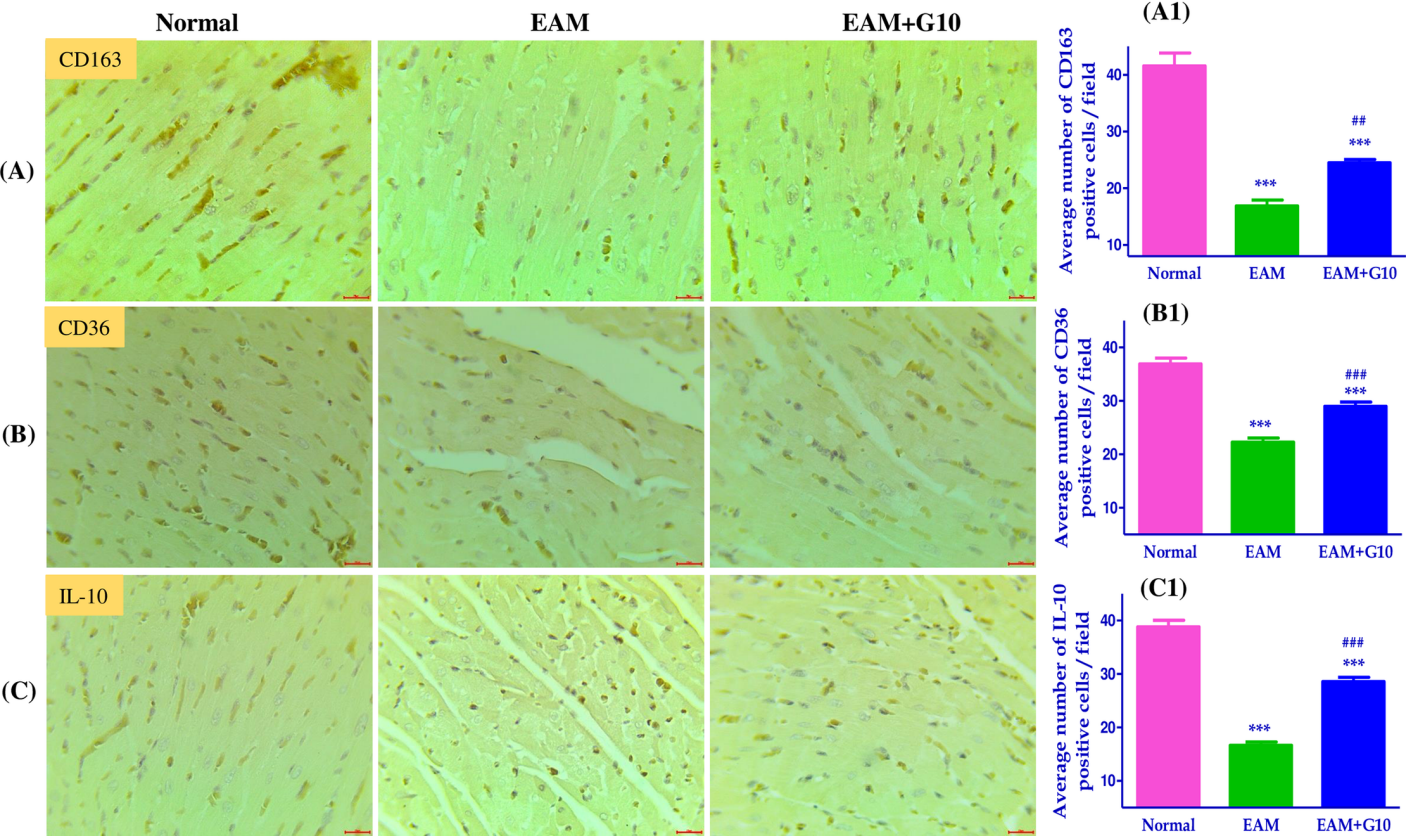
Effect of gallein on M2-like macrophage phenotype in the heart of EAM rats. IHC staining for (A-A1) CD163, (B-B1) CD36, (C-C1) IL-10 and their quantification data. Scale bar = 20 μm. Each bar represents mean ± SEM, n = 4–5. Normal, age matched normal rats; EAM, rats with experimental autoimmune myocarditis treated with vehicle; EAM+G10, rats with EAM treated with gallein. ***p<0.001 vs Normal; ^###^p<0.001 and ^##^p<0.01 vs EAM.

IHC staining showed that the M2 phenotype markers CD163, CD36, and IL-10 positive cells were significantly downregulated in the heart sections of EAM rats. In contrast gallein treatment significantly up-regulated the numbers of CD163, CD36 and IL-10 positive cells in EAM rats, when compared with vehicle treated EAM rats (Fig 6A–6C).

### Effects of gallein on human macrophage polarization

**Fig 7:**
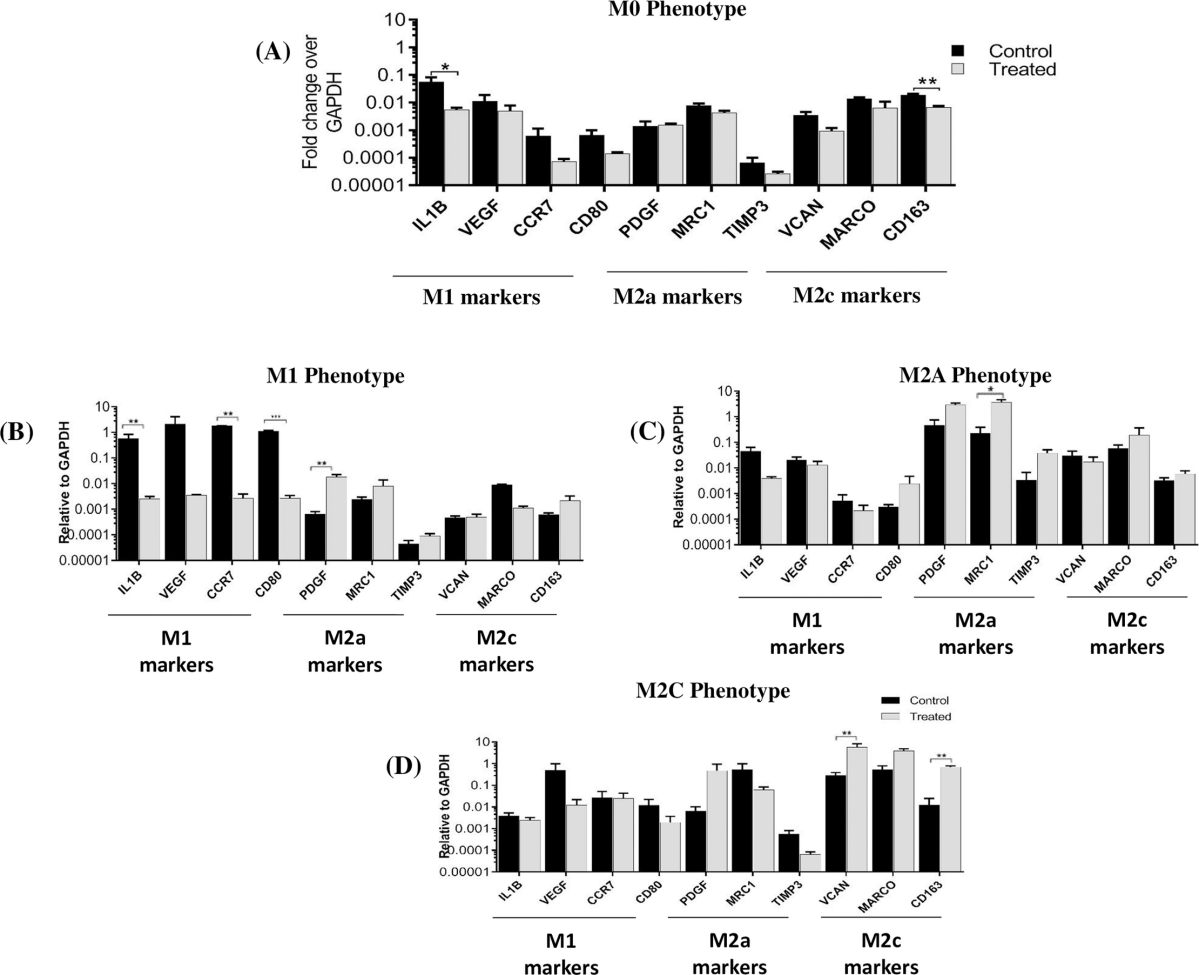
Effect of gallein on human M1 and M2 macrophages in vitro. (A-D) Changes in gene expression of macrophage phenotype markers after gallein treatment (qRT-PCR from n = 3 experimental replicates). Data are normalized to expression of GAPDH. Control groups in each phenotype are cells without gallein treatment. Data are shown as Mean ± SEM. *p<0.05, **p<0.01, ***p<0.001 vs Control.

Next, we investigated whether gallein would directly modulate M1 and M2 polarization in primary human macrophages *in vitro*. We polarized primary human macrophages to three distinct phenotypes (M1, M2a, and M2c), as well as an unactivated control (M0). Gene expression of different macrophage phenotype markers in M0 macrophages (unactivated) with or without gallein treatment are shown in (Fig 7A). Our results showed that M1 markers were suppressed in the presence of gallein when normalized to GAPDH (Fig 7B), and to M0, (S1 Fig), although this finding was only significant for IL1B, suggesting that gallein inhibits the pro-inflammatory M1 phenotype. These results were more pronounced for M1 macrophages (Fig 7B), with gallein significantly inhibiting expression of all M1 markers evaluated. In addition, gallein treatment increased expression of the M2 markers PDGFB in M1 macrophages, MRC1 in M2a macrophages (Fig 7C), and VCAN and CD163 in M2c macrophages (Fig 7D).

## Discussion

In this work, we found that 1) GRK2 expression is increased in the human failing heart, with simultaneous activation of M1 and decrease in M2 macrophage polarization; 2) the small molecule Gβγ inhibitor, gallein, sharply suppresses M1-like and promotes M2-like macrophage phenotype in a rat model of EAM and in human macrophages *in vitro*; and 3) gallein treatment alleviates cardiac dysfunction, fibrosis, and infiltration of inflammatory and mast cells.

Extensive clinical and experimental data suggest that a number of small molecules bind to Gβγ to modulate its effector interaction [25]. In addition, they could inhibit several Gβγ dependent signaling events and GRK2-Gβγ interaction [21]. In our previous study, we found that inhibition of GRK2-Gβγ interactions via treatment with gallein leads to amelioration of HF[1]. Previous studies showed that LV GRK2 levels were increased in patients with idiopathic cardiomyopathy, volume overload, cardiac ischemia, and LV hypertrophy [25]. Moreover, in some animals the development of overt HF was preceded by an elevation of GRK2 levels that correlates with progressive impaired myocardial contractility [26]. In line with this, our data also indicate that levels of GRK2 are highly upregulated in myocardium of patients with cardiac failure. All of these findings strongly suggest that the Gβγ-GRK2 interaction contributes to cardiac dysfunction and support the idea that Gβγ signaling offers a possible therapeutic target. In this study, we tested whether the Gβγ small molecule inhibitor gallein could be used to target Gβγ in the myocardium and thereby improve cardiac function via interaction with GRK2 in a myosin-induced rat model of EAM.

Activated Gβγ signaling directly triggers STAT4 and IFNγ production, which influences macrophage infiltration and polarization. Production of STAT4 and IFNγ triggers inflammation as well as increases Th1 and decreases Th2 phenotype in T cell receptor (TCR) stimulated human CD4^+^ Th cells [13]. Progression of HF was correlated with the accumulation of M1 macrophage phenotype in the myocardium and increased production of M1 macrophages, associated with production of proinflammatory cytokines such as IFNγ, IL-6, and TNFα, which all lead to cardiac inflammation [27, 28]. In the present study, the number of M1 macrophages accumulated in myocardium with upregulation of proinflammatory factors and cytokines expression in myosin-induced EAM rats, whereas treatment with gallein significantly attenuated M1 macrophage markers, cytokines and proinflammatory factor expression in the hearts of rats with EAM. Next, we confirmed M1 phenotype markers levels in human peripheral blood monocyte-derived macrophages by qRT-PCR analysis. We have observed that LPS/IFNγ-induced M1 macrophages and gene expression of M1-associated markers were significantly suppressed by gallein treatment *in vitro*. These findings clearly suggest that the small molecule Gβγ inhibitor, gallein, effectively modulates the M1 phenotype and its associated proinflammatory factors and cytokine levels in myosin induced EAM heart and LPS/IFNγ-induced human macrophages.

Previous studies have demonstrated that proinflammatory cytokines and M1 macrophages skew the secretion of HMGB1 [14, 28]. Translocation of HMGB1 from the nucleus to the cytoplasm or to the extracellular space acts as an alarmin, an endogenous molecule that evokes an immune response or proinflammatory cytokine secretion, and extracellular HMGB1 is involved in the pathogenesis of HF [29]. Our previous data demonstrated that HMGB1 could directly promote cardiac remodeling in aging mice [28]. Moreover, HMGB1 contributed to cardiac fibroblast proliferation, migration, and collagen deposition, leading to EAM progression [30]. Recently, a study reported that HMGB1 facilitated macrophage programming towards a proinflammatory M1-like phenotype in an EAM model, while antagonism of HMGB1 reduced cardiac damage [31]. Furthermore, activation of HMGB1-mediated inflammatory response could be modulated through TLR4. HMGB1 interaction with TLR4 triggers downstream signaling activation [28]. In our study HMGB1 and TLR4 expression were upregulated in the heart of EAM rats, whereas treatment with gallein attenuated these changes. It is plausible to speculate that HMGB1 might have interacted with TLR4 to produce cardiac inflammation in EAM rats.

Extracellular HMGB1 binding with TLR4 activates ERK/NFκB signaling and has an important role in inflammation. Increasing evidence suggests that ERK levels are dramatically increased during cardiac damage, as shown in an ischemic pig model and in the human heart [32]. In addition, activation of ERK seems to be a causal factor for cardiac inflammation by HMGB1, and phosphorylated ERK has been associated with the macrophage polarization and inflammatory process [33]. Here we observed that gallein treatment effectively attenuated the phosphorylation of ERK1/2 in the hearts of EAM rats. These findings confirm that M1-like macrophages might activate HMGB1 signaling to induce cardiac inflammation in EAM rats, and that treatment with the small molecule Gβγ inhibitor gallein can modulate this signaling pathway in EAM rats.

IL-4 regulates M2 macrophage polarization via the ERK signaling pathway to protect against atherosclerosis [34]. The M2 macrophage is induced by IL-4/IL-13, IL-10, and glucocorticoids. Up-regulated M2 subtype improves cardiac function, and suppresses the cardiac inflammation; also, it plays a crucial functional role the repair phase of myocarditis [15]. These earlier findings suggest that the M2 phenotype plays a substantial role in HF. Interestingly, we found that M2 phenotype levels were significantly downregulated in EAM hearts, whereas treatment with gallein restored their levels. Native monocytes, in the presence of cytokines such as IL-4/IL-13 or IL-10 can be primed towards M2a and M2c phenotypes. Therefore, we tested if gallein could affect polarization of such anti-inflammatory/reparative human M2 phenotype M2a and M2c. Interestingly, gallein upregulated M2a and M2c phenotype markers in IL-4/IL-13- and IL-10-treated human macrophages *in vitro*. These results suggest that gallein ameliorates cardiac dysfunction and inflammation by inhibiting M1 polarization and promoting M2 polarization in rats with myosin induced EAM hearts and human macrophages. Thus, it appears that the reprogramming of macrophages towards the M2 phenotype contributes to protection against cardiac damage and heart failure.

## Conclusions

In summary, we have demonstrated that the small molecule Gβγ inhibitor gallein functions *in vivo* to improve cardiac function and halts HF progression in rat model of EAM. This small molecule Gβγ inhibitor effectively modulated GRK2, proinflammatory cytokines, and M1 macrophage polarization with simultaneous restoration of M2 phenotype in the EAM heart. Our data also confirm the potential therapeutic role of gallein in human M1 and M2 macrophages in vitro. Gallein treatment effectively suppressed the M1 phenotype and enhanced the M2a and M2c phenotypes in human macrophages. Although further studies are needed to understand the effect of gallein on phenotypic reprogramming from M1 to M2, results from our studies suggest that a small molecule Gβγ inhibitor may be an effective therapeutic paradigm for the treatment of HF.

## Supporting information

S1 Fig Effect of gallein on human M1 and M2 macrophage phenotype. (A-C) Gene expression changes of known markers in macrophages phenotypes normalized with M0 phenotypes (qRT-PCR from n = 3 experimental replicates). Control groups are cells without gallein treatment. Data are shown as Mean ± SEM. *p<0.05, **p<0.01, ***p<0.001.
(PPTX)
