# Supplementary material for: Small molecule disruption of G protein βγ subunit signaling reprograms human macrophage phenotype and prevents autoimmune myocarditis in rats
Source: PLoS One. 2018 Jul 19;13(7):e0200697. doi: 10.1371/journal.pone.0200697 (PMC6053176; doi:10.1371/journal.pone.0200697)
Supplement: S1 Fig — (A-C) Gene expression changes of known markers in macrophages phenotypes normalized with M0 phenotypes (qRT-PCR from n = 3 experimental replicates). Control groups are cells without gallein treatment. Data are shown as Mean ± SEM. *p<0.05, **p<0.01, ***p<0.001. (PPTX) [file pone.0200697.s001.pptx]

## Slide 1
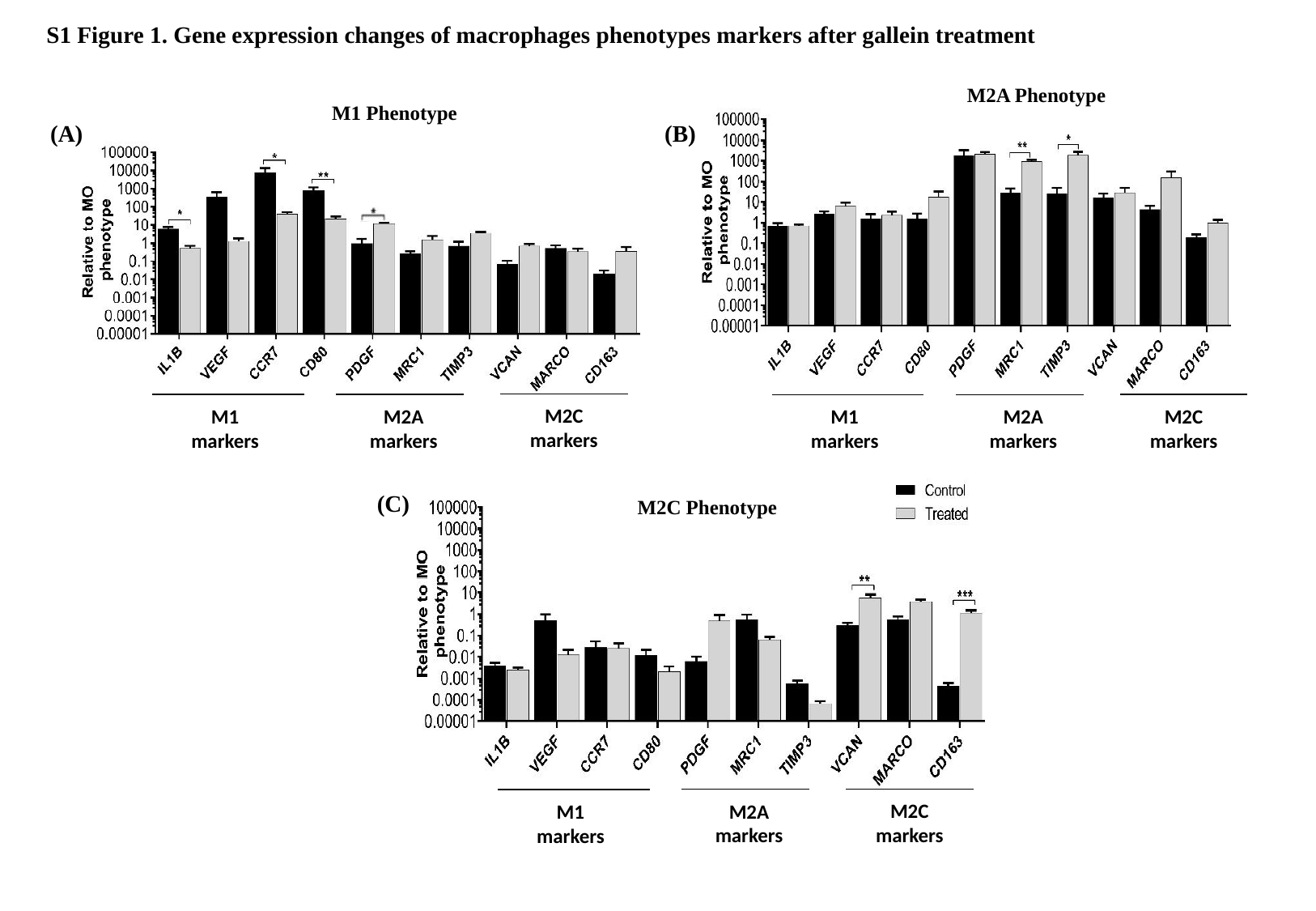

S1 Figure 1. Gene expression changes of macrophages phenotypes markers after gallein treatment
M2A Phenotype
M1 Phenotype
M2C
markers
M2A
markers
M1 markers
(A)
(B)
M2C
markers
M2A
markers
M1 markers
M2C Phenotype
(C)
M2C
markers
M2A
markers
M1 markers
